# Upper Respiratory Infections in Schools and Childcare Centers Reopening after COVID-19 Dismissals, Hong Kong

**DOI:** 10.3201/eid2705.210277

**Published:** 2021-05

**Authors:** Min Whui Fong, Nancy H.L. Leung, Benjamin J. Cowling, Peng Wu

**Affiliations:** WHO Collaborating Centre for Infectious Disease Epidemiology and Control, University of Hong Kong, Hong Kong, China (M.W. Fong, N.H.L. Leung, B.J. Cowling, P. Wu);; Laboratory of Data Discovery for Health Limited, Hong Kong (B.J. Cowling, P. Wu)

**Keywords:** acute respiratory illness, coronavirus disease, COVID-19, outbreaks, respiratory infections, rhinovirus, school health, schools, upper respiratory tract infection, URTI, viruses, SARS-CoV-2, severe acute respiratory syndrome coronavirus 2

## Abstract

A large number of common cold outbreaks in Hong Kong schools and childcare centers during October–November 2020 led to territorywide school dismissals. Increased susceptibility to rhinoviruses during prolonged school closures and dismissals for coronavirus disease and varying effectiveness of nonpharmaceutical interventions may have heightened transmission of cold-causing viruses after school attendance resumed.

Many countries implemented school closures and dismissals in 2020 as a public health measure to reduce spread of coronavirus disease (COVID-19), caused by severe acute respiratory syndrome coronavirus 2 (SARS-CoV-2). In Hong Kong, schools were dismissed after the Lunar New Year holiday in late January 2020 and remained dismissed until late May; during early July–late September, schools were dismissed again in response to a surge in cases of COVID-19 (Figure, panel A). During the dismissal periods, most school campuses remained open to staff but lessons were delivered online. Here, we report a large number of outbreaks of acute upper respiratory tract infections (URTIs), likely rhinovirus infections, that were identified during October–November 2020 in reopened primary schools, secondary schools, kindergartens, childcare centers, and nursery schools in Hong Kong; these outbreaks led to further territorywide school dismissals for younger children. 

**Figure:**
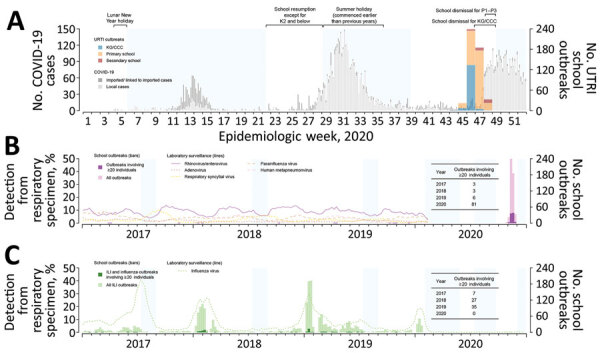
Respiratory illness outbreaks in primary and secondary schools, kindergartens, childcare centers, and nursery schools in Hong Kong. A) Weekly number of outbreaks of upper respiratory tract infection in schools reported during October 25–November 28, 2020, overlaid on the epidemic curve of daily COVID-19 case numbers in Hong Kong, by date of reporting. B) Weekly numbers of outbreaks of upper respiratory tract infection in schools during weeks 44–48 of 2020 and school outbreaks involving >20 persons reported during 2017–2020. Lines indicate detection rates of rhinovirus/enterovirus and other viruses in respiratory specimens collected for laboratory surveillance. C) Weekly numbers of outbreaks of influenza-like illness and influenza in schools reported during 2017–2020. Dotted line indicates detection of influenza virus in respiratory specimens collected for laboratory surveillance. Durations of territorywide regular school breaks (summer holiday) during 2017–2019 and school dismissals implemented in response to COVID-19 in 2020 are shaded in blue. ILI, influenza-like-illness; URTI, upper respiratory tract infection; KG/CCC, kindergartens, child-care centers, and nursery schools; K2, kindergarten year 2 (4–5 years of age); P1–P3, primary school years 1–3 (6–9 years of age)

In the last week of October 2020, the Hong Kong Centre for Health Protection began receiving reports of URTI outbreaks in kindergartens, childcare centers, nursery schools, and primary schools. Outbreaks of URTIs in schools continued to increase rapidly in the following weeks (Figure, panel A). A school URTI outbreak was defined as >3 students in the same class each developing >2 symptoms of respiratory tract infection within 4 days (Table). Various measures were implemented in response to these URTI outbreaks. Initially, schools with outbreaks were advised to dismiss affected classes for >3 days; this guideline was expanded to dismissal of entire schools for >7 days beginning November 18. SARS-CoV-2 testing was also conducted for students in affected classes and all staff in these schools. 

**Table:** Symptoms reported in 81 upper respiratory tract infection outbreaks involving 20 persons in schools, kindergartens, childcare centers, and nursery schools, Hong Kong, October–November 2020

Symptoms	No. (%) outbreaks
Cough, runny nose, fever, and sore throat	49 (60.5)
Cough, runny nose, and sore throat	27 (33.3)
Cough, runny nose, and fever	1 (1.2)
Cough and runny nose	4 (4.9)

Territorywide school dismissals took effect beginning November 14. Kindergartens, childcare centers, and nursery schools were dismissed first, for 2 weeks, because most outbreaks had occurred in this age group (*1*); primary grades 1–3 were dismissed beginning November 23. In total, 482 outbreaks were reported during October 25–November 28, including 308 (63.9%) outbreaks in primary schools, 149 (30.9%) in kindergartens, childcare centers, and nursery schools, and 25 (5.2%) in secondary schools (*2*). There were 81 larger outbreaks involving >20 persons (*3*), equal to the total number of 2017–2019 outbreaks of the same scale for URTIs (Figure, panel B), and influenza-like illnesses and influenza (Figure, panel C). Laboratory testing suggested that rhinoviruses or enteroviruses were the likely pathogens, and no SARS-CoV-2 or influenza viruses were detected (*1*). It is very unusual for schools to be closed or dismissed in response to outbreaks of common colds. In this particular circumstance, one rationale for dismissing students was to spare the public health laboratory resources needed to test the many samples from school outbreaks for SARS-CoV-2, despite the very low risk of in-school transmission (*4*). 

From cross-sectional surveys conducted in February and March 2020, we reported that 75% of school-aged children did not have contact with persons outside their own household when schools were dismissed (*5*). Indicators of respiratory virus activity, such as rates of consultation for influenza-like illnesses and detection of influenza viruses in respiratory specimens, remained extremely low throughout 2020 (*6*). However, population susceptibility to rhinoviruses and other respiratory viruses, including influenza viruses, might have been increasing over time because persons were likely less exposed to the viruses when intense social distancing measures, including school dismissals, were implemented in response to the COVID-19 pandemic. This would have increased transmission potential when schools resumed. In England in September 2020, ≈2 weeks after full reopening of schools following prolonged dismissals, a substantial increase in the detection of rhinoviruses among adults was recorded (*7*), possibly driven by transmission among children. 

URTI outbreaks caused by the respiratory viruses responsible for common colds (i.e., other than influenza viruses) occurred in Hong Kong schools despite a wide range of infection control measures being in place. Staff and students wore face masks at all times; lunch hours were cancelled, desks were spaced out, and group activities were limited (*4*). Although in general transmission modes may be similar for different respiratory viruses, how much each mode contributes to transmission of a specific virus remains unclear; therefore, the effectiveness of certain nonpharmaceutical interventions might differ between viruses (*8*). For example, face masks were shown to be efficacious in blocking the release of coronaviruses and influenza viruses, but not rhinoviruses, in exhaled breath (*9*). In addition, enveloped viruses (e.g., coronaviruses and influenza viruses) are less resistant to lipophilic disinfectants than nonenveloped viruses (e.g., rhinoviruses) (*10*). This difference might have played a role in URTI outbreaks in Hong Kong related to rhinoviruses but not influenza viruses, even though individual persons and schools had practiced frequent cleaning and disinfection. Our findings highlight the increased risk posed by common cold viruses in locations where schools have been closed or dismissed for extended periods during the COVID-19 pandemic. 

## References

[1] Government of the Hong Kong Special Administrative Region. Media session (2020 November 12) [cited 2020 Dec 23]. https://isd.wecast.hk/vod/?id=11384

[2] Government of the Hong Kong Special Administrative Region. Media session (2020 November 20) [cited 2020 Dec 23]. https://isd.wecast.hk/vod/?id=11436

[3] Centre for Health Protection. Press releases [cited 2020 Dec 23]. https://www.chp.gov.hk/en/media/116/index.html.

[4] Fong MW, Cowling BJ, Leung GM, Wu P. Letter to the editor: COVID-19 cases among school-aged children and school-based measures in Hong Kong, July 2020. Euro Surveill. 2020;25:2001671. 10.2807/1560-7917.ES.2020.25.37.2001671 PMC7502885 32945255

[5] Cowling BJ, Ali ST, Ng TWY, Tsang TK, Li JCM, Fong MW, et al. Impact assessment of non-pharmaceutical interventions against coronavirus disease 2019 and influenza in Hong Kong: an observational study. Lancet Public Health. 2020;5:e279–88. 10.1016/S2468-2667(20)30090-6 32311320 PMC7164922

[6] Centre for Health Protection. Flu express. 2020 Dec 20–26 [cited 2021 Jan 19]. https://www.chp.gov.hk/files/pdf/fluexpress_week52_31_12_2020_eng.pdf

[7] Poole S, Brendish NJ, Tanner AR, Clark TW. Physical distancing in schools for SARS-CoV-2 and the resurgence of rhinovirus. Lancet Respir Med. 2020;8:e92–3. 10.1016/S2213-2600(20)30502-6 33289636 PMC7581315

[8] Leung NHL. Transmissibility, transmission and its control of respiratory viruses. Nat Rev Microbiol. 2021;22:1–18. 10.1038/s41579-021-00535-6 PMC7982882 33753932

[9] Leung NHL, Chu DKW, Shiu EYC, Chan KH, McDevitt JJ, Hau BJP, et al. Respiratory virus shedding in exhaled breath and efficacy of face masks. Nat Med. 2020;26:676–80. 10.1038/s41591-020-0843-2 32371934 PMC8238571

[10] Lin Q, Lim JYC, Xue K, Yew PYM, Owh C, Chee PL, et al. Sanitizing agents for virus inactivation and disinfection. VIEW. 2020;1:e16. 10.1002/viw2.16 PMC7267133 34766164

